# P-635. “Exploring Knowledge and Barriers to HPV Vaccination among Mothers of 9-14 Years Olds - a Descriptive Study in a Tertiary Care Hospital”

**DOI:** 10.1093/ofid/ofae631.832

**Published:** 2025-01-29

**Authors:** Tara B Nair, Dr Shiju Kumar C, Dr B S Vishnu, Dr Lizy Vincent

**Affiliations:** Kims Trivandrum, Trivandrum, Kerala, India; Kimshealth, Trivandrum, Kerala, India; Kimshealth, Trivandrum, Kerala, India; Kimshealth, Trivandrum, Kerala, India

## Abstract

**Background:**

Human Papillomavirus (HPV) vaccination is pivotal in preventing cervical cancer and other HPV-related diseases. However, uptake remains suboptimal, partly due to parental knowledge gaps and barriers

Association between Knowledge Level & Factors Hindering Vaccination

**Figure:**
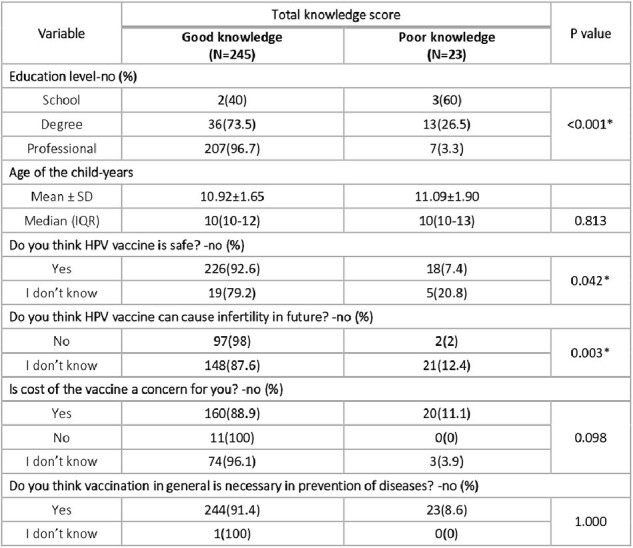

**Methods:**

This prospective descriptive study was conducted in a tertiary care hospital in Trivandrum, Kerala. A structured preformed questionnaire was administered to total of 268 participants who were mothers of girl children aged 9-14 years attending outpatient pediatric department. The questionnaire assessed knowledge about HPV, the vaccine available for HPV, and factors influencing vaccinations.

**Results:**

Among the 268 study subjects, a significant majority, comprising 91.4%, demonstrated a commendable level of understanding regarding the HPV vaccine. Various factors were identified as barriers to vaccination uptake, with safety concerns being the most prevalent, cited by 91% of respondents. Additionally, a notable percentage (36.9%) expressed uncertainty regarding potential future infertility concerns linked to the vaccine, while 67.2% cited the high costs associated with it as a deterrent. Nevertheless, an overwhelming majority (99.6%) acknowledged the indispensable role of vaccines in disease prevention.

**Conclusion:**

The substantial knowledge observed within our study cohort can be credited to the state's commendable literacy rates and the concerted efforts of governmental and non-governmental entities to fortify healthcare awareness. This study provides valuable insights the gaps in knowledge and measures to augment HPV vaccination rates among adolescents.

**Disclosures:**

**All Authors**: No reported disclosures

